# Prevention of Bone Destruction by Mechanical Loading Is Not Enhanced by the Bruton’s Tyrosine Kinase Inhibitor CC-292 in Myeloma Bone Disease

**DOI:** 10.3390/ijms22083840

**Published:** 2021-04-07

**Authors:** Fani Ziouti, Maximilian Rummler, Beatrice Steyn, Tobias Thiele, Anne Seliger, Georg N. Duda, Bjarne Bogen, Bettina M. Willie, Franziska Jundt

**Affiliations:** 1 Department of Internal Medicine II, University Hospital Würzburg, 97080 Würzburg, Germany; fziouti@gmail.com; 2 Research Centre, Shriners Hospital for Children-Canada, Montreal, QC H4A 0A9, Canada; maximilian.rummler@mail.mcgill.ca (M.R.); beatrice.steyn@mail.mcgill.ca (B.S.); 3 Department of Pediatric Surgery, McGill University, Montreal, QC H4A 3J1, Canada;; 4 Julius Wolff Institute and Berlin Institute of Health Center for Regenerative Therapies, Charité−Universitätsmedizin Berlin, 13353 Berlin, Germany; tobias.thiele@charite.de (T.T.); anne.seliger@charite.de (A.S.); georg.duda@charite.de (G.N.D.); 5 Institute of Clinical Medicine, University of Oslo and Department of Immunology, Oslo University Hospital, 0424 Oslo, Norway; bjarne.bogen@medisin.uio.no; 6 Comprehensive Cancer Center Mainfranken, 97080 Würzburg, Germany

**Keywords:** multiple myeloma, cancer-induced bone disease, Bruton’s tyrosine kinase inhibitor CC-292, skeletal mechanobiology, bone adaptation, mechanical loading

## Abstract

Limiting bone resorption and regenerating bone tissue are treatment goals in myeloma bone disease (MMBD). Physical stimuli such as mechanical loading prevent bone destruction and enhance bone mass in the MOPC315.BM.Luc model of MMBD. It is unknown whether treatment with the Bruton’s tyrosine kinase inhibitor CC-292 (spebrutinib), which regulates osteoclast differentiation and function, augments the anabolic effect of mechanical loading. CC-292 was administered alone and in combination with axial compressive tibial loading in the MOPC315.BM.Luc model for three weeks. However, neither CC-292 alone nor its use in combination with mechanical loading was more effective in reducing osteolytic bone disease or rescuing bone mass than mechanical stimuli alone, as evidenced by microcomputed tomography (microCT) and histomorphometric analysis. Further studies are needed to investigate novel anti-myeloma and anti-resorptive strategies in combination with physical stimuli to improve treatment of MMBD.

## 1. Introduction

Multiple myeloma (MM) is an incurable plasma cell-derived neoplasia which currently accounts for 10% of all hematologic malignancies [1]. MM largely affects the elderly, with a median age of 69 years at diagnosis [2]. It is characterized by aberrant proliferation of monoclonal plasma cells in the bone marrow and large amounts of secreted monoclonal immunoglobulins in the serum and/or urine. Over 80% of the patients with MM develop bone disease [3], which is detected by conventional skeletal survey, whole-body computed tomography (CT), positron emission tomography (PET)/CT, and magnetic resonance imaging (MRI) [4]. Among the most frequent skeletal-related events are pain and pathologic fractures, affecting morbidity and mortality of MM patients [5]. MM bone disease (MMBD) occurs when MM cells disrupt the intricate balance between osteoclasts and osteoblasts, triggering osteolytic bone destruction [6]. This leads to generalized osteopenia and/or the formation of characteristic “punched-out” lesions. The pathogenetic mechanisms of MMBD are yet to be completely understood, and there is a need to develop early and alternative treatment strategies [7,8].

Current pharmacological treatment strategies for MMBD include the use of bisphosphonates and denosumab, a receptor activator nuclear factor kappa-B (RANK) ligand inhibitor, and focus on limiting bone resorption [6]. Novel drugs are under investigation in pre-clinical MM models for their use in MMBD. Bruton’s tyrosine kinase (BTK) regulates osteoclast differentiation by linking RANK and immunoreceptor tyrosine-based activation motif signals [9]. As a result, calcium signaling is activated and the key transcription factor for osteoclast differentiation, nuclear factor of activated T cells 1, is induced [9]. In a mouse xenograft human MM model, a combination of the BTK inhibitor CC-292 (spebrutinib, Celgene Corporation) with the proteasome inhibitor carfilzomib increased vertebral trabecular bone mass compared to carfilzomib alone and reduced tumor burden [10]. While CC-292 does not inhibit osteoclast differentiation, it inhibits the formation of the osteoclast sealing zone, thus interfering with osteoclast function and activity [10], and leading to reduced serum levels of the bone resorption biomarker carboxy-terminal collagen cross-linking telopeptide in mice [10] and humans [11]. CC-292 reduced tumor load and normalized tumor-associated expansion of T cells and monocytes, and did not affect T cell function in the adoptive transfer TCL1 mouse model of chronic lymphocytic leukemia [12]. Similarly, the oral and selective BTK inhibitor PCI-32765 (ibrutinib) inhibits MM cell growth and MM cell-induced osteolysis of implanted human bone chips in severe combined immunodeficient (SCID) mice [13]. Interestingly, a Phase I study showed that CC-292 administration resulted in dose-dependent responses in relapsed/refractory chronic lymphocytic leukemia patients, but despite achieving high BTK receptor occupancy, its clinical activity was inferior to ibrutinib or acalabrutinib [14]. 

In addition to limiting bone resorption, stimulating bone formation is a major treatment goal in MMBD. Inhibition of sclerostin and dickkopf 1 (DKK1) are promising anabolic treatment approaches, as preclinical studies have shown increased bone mass and decreasing osteolysis in pre-clinical models of MMBD [15,16,17]. Moreover, it has been demonstrated in rodents and non-human primates that simultaneous inhibition of sclerostin and DKK1 leads to synergistic bone formation, highlighting these proteins as potential targets in the treatment of MMBD [18]. 

It is known that mechanical loading leads to reductions in osteocyte expression ofsclerostin and DKK1, which contribute to the bone formation response through activation of WNT target genes [19,20]. In pre-clinical MM models, we and others have shown that physical stimuli counteract the deleterious effects of MM on the skeleton, increase bone formation, and even reduce tumor growth [21,22]. We applied axial compressive tibial loading in the syngeneic MOPC315.BM.Luc MMBD model [22], which we established as a platform for the biomaterial characterization of the mineralized and nonmineralized matrix in the cortical bone in young and skeletally mature BALB/c mice [7,23,24]. Mechanical stimulation increases bone mass by upregulating osteoblast activity while reducing osteoclast activity simultaneously [19,20,25,26]. Physical stimuli have a potent non-pharmacological anabolic effect as exercise enhances bone mass in the elderly [27,28]. Recently, we showed in a clinical study that whole-body vibration exercise is beneficial with respect to physical performance in patients with monoclonal gammopathy of undetermined significance, a precursor condition of MMBD [29].

In this study, we hypothesized that prevention of bone destruction and increase in bone mass by mechanical loading are enhanced by the BTK inhibitor CC-292. We injected syngeneic MOPC315.BM.Luc cells into the proximal tibia of BALB/c mice. Mice underwent in vivo axial compressive loading of the tibia [30] and were treated with CC-292 for three weeks. Changes in bone microstructure were assessed by microcomputed tomography (microCT) imaging every fifth day starting at day 14 day until day 33 after tumor inoculation.

## 2. Results

### 2.1. CC-292 Does Not Prevent Osteolytic Bone Destruction in NonLoaded Mice

**Figure 1:**
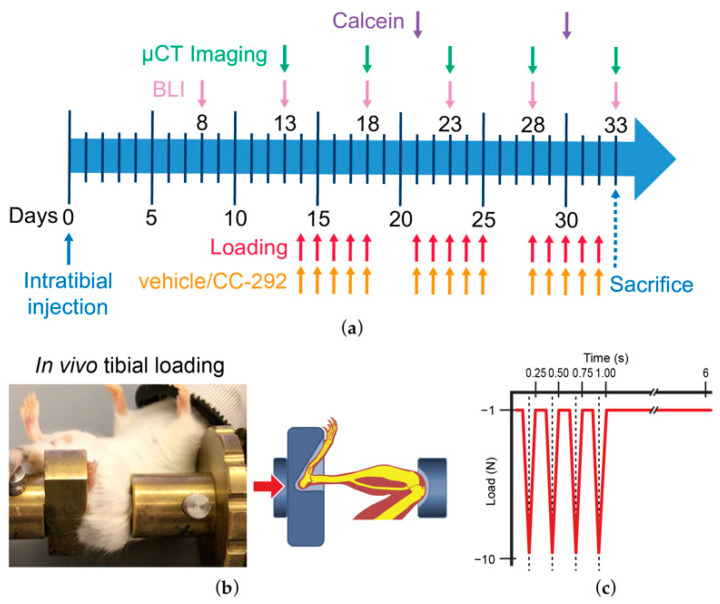
Overview of the loading experiment. (**a**) The experimental timeline. Mice were injected with MOPC315.BM.Luc cells on day 0; after 8 days, bioluminescence imaging (BLI) started; on day 13, microCT imaging started; on day 14, the loading regime and the vehicle and CC-292 oral treatment began; mice were sacrificed on the last microCT day, day 33. (**b**) Balb/c mouse undergoing loading. The knee and the ankle are fixated and loading is applied. The head lies in an anesthetic mask, through which oxygen and isoflurane are supplied. (**c**) Schematic overview of the applied loading cycles. Four peak loads of −10 N were applied using a 4 Hz frequency, after which a 5 s pause was inserted. Then, the cycle started anew. Cycles were repeated 216 times a day, which lasted approximately 5 min.

Mice were intratibially injected either with phosphate-buffered saline (PBS) or MOPC315.BM.Luc cells on day 0. After two weeks, the loading regime as well as the vehicle and CC-292 oral treatment began and lasted for three weeks (Figure 1).

**Figure 2:**
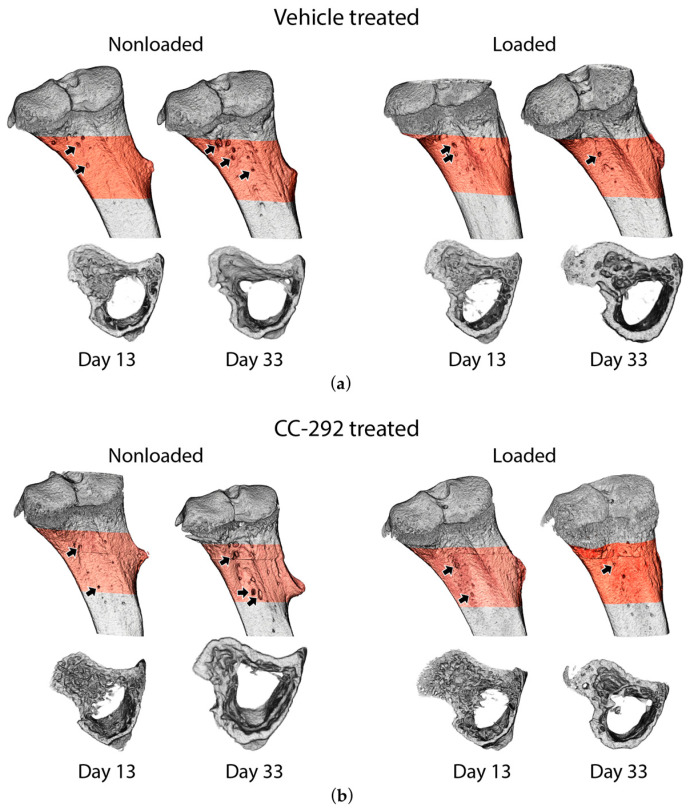
3D renderings of the metaphyseal region of a nonloaded and a loaded tibia of vehicle and CC-292 treated MM-injected mice, respectively. (**a**) Representative 3D renderings of vehicle treated mice are depicted at days 13 and 33 of the experiment. (**b**) Representative 3D renderings of CC-292 treated mice are depicted as well. The arrows mark osteolytic lesions through the cortex. The volume of interest (VOI) for microCT analysis is marked in red.

**Figure 3:**
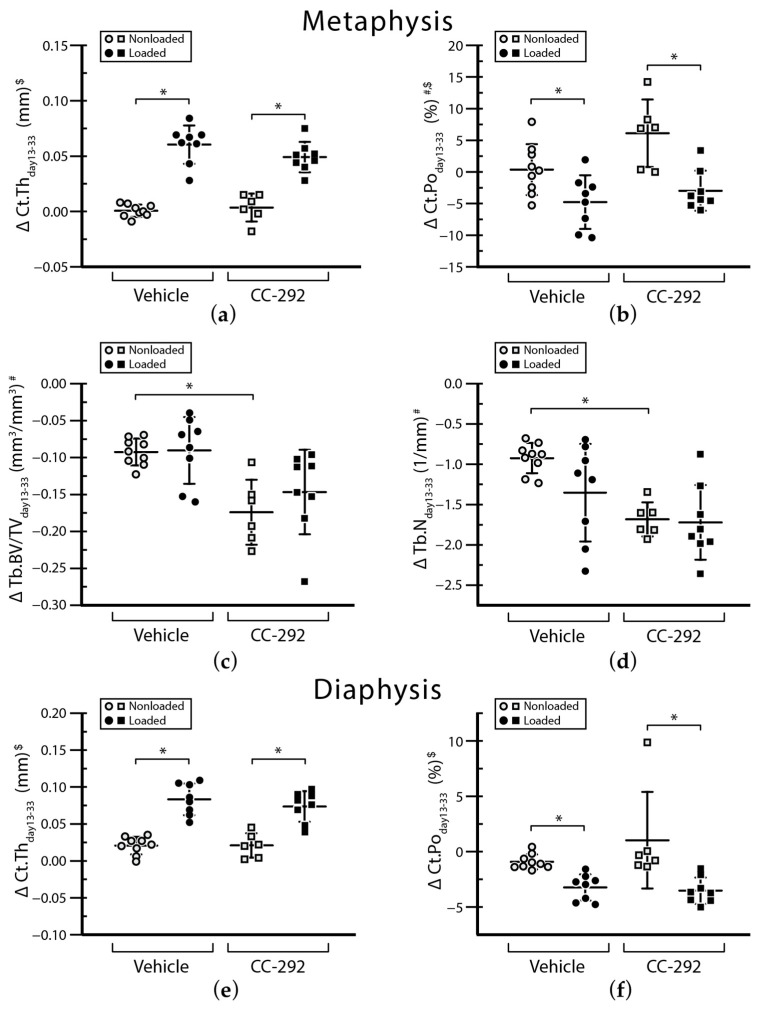
MicroCT parameters between day 13 and 33 of the experiment. The graphs depict cortical and trabecular microarchitectural parameters in the metaphysis and diaphysis. Shown are changes between day 13 and day 33 of (**a)** metaphyseal cortical thickness, (**b**) metaphyseal cortical porosity, (**c**) trabecular bone volume fraction, (**d**) trabecular number, (**e**) diaphyseal cortical thickness, and (**f**) diaphyseal cortical porosity. Individual values are given together with mean ± SD. ANOVA main effects: (#) treatment, ($) loading, followed by Tukey–Kramer post-hoc test, * *p* < 0.05.

After MM injection, bioluminescence imaging (BLI) signals in all mice were detected already at day 8 and local tumor growth was confirmed (data not shown). MicroCT three-dimensional (3D) renderings of MM-injected tibiae at day 13 compared to day 33 revealed an increase in the number and size of osteolytic cortical lesions in all nonloaded mice (Figure 2a,b, left panels). Quantification of in vivo microCT cortical microstructural parameters at the metaphyseal region shows changes over the experimental period (Δ between day 13 and day 33, Figure 3a,b, Supplementary Tables S1 and S2), that reflect the osteolytic potential of the MOPC315.BM.Luc cells in young animals that are still growing.

**Figure 4:**
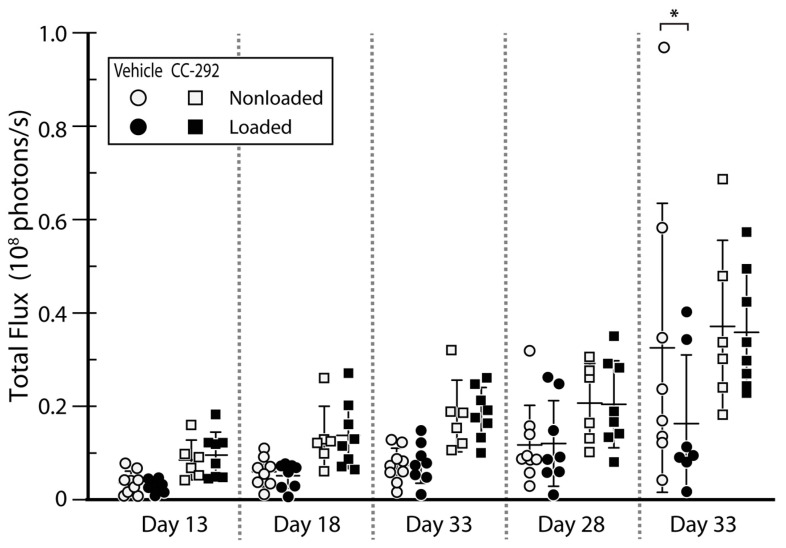
BLI analysis of vehicle and CC-292 treated mice. BLI signals (total flux in photons/sec) of MM-injected mice treated with vehicle (circles) or CC-292 (squares), nonloaded (white), and loaded (gray) at days after inoculation (mean ± SD). Two-way ANOVA followed by Tukey–Kramer post-hoc test, * *p* < 0.05.

**Figure 5:**
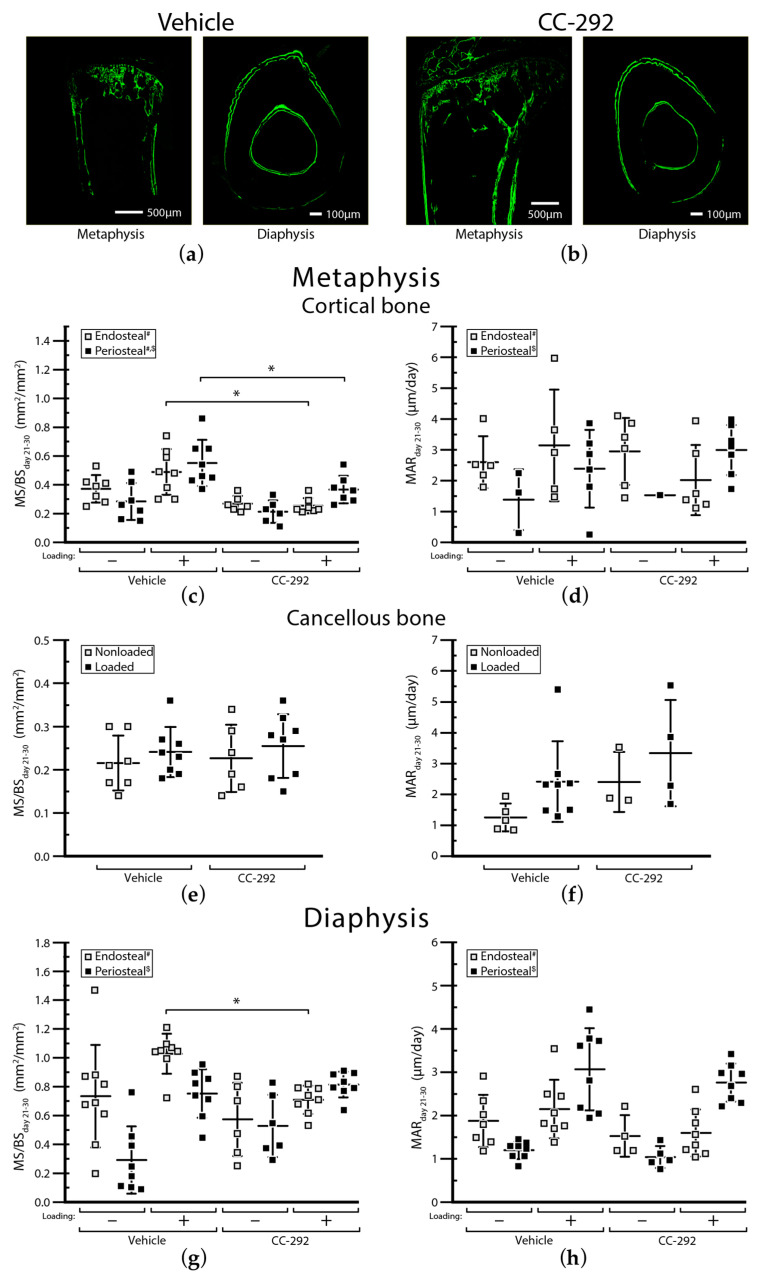
Conventional histomorphometry. Shown (**a**,**b**) are representative images of calcein-labeled sections of the metaphysis (upper) or diaphysis (lower) of either vehicle or CC-292 treated mice. Plotted are (**c**) mineralized surface area (MS/BS) and (**d**) mineral apposition rate (MAR) in metaphyseal cortical bone, (**e**) MS/BS and (**f**) MAR in metaphyseal cancellous bone, and (**g**) MS/BS and (**h**) MAR in diaphyseal bone between days 21 and 30 measured in the ROI at either the endosteal or the periosteal surface in all groups (vehicle, CC-292) either nonloaded (−) or loaded (+). Plots show individual values together with mean ± SD. ANOVA main effects: (#) treatment, ($) loading, * *p* < 0.05.

BLI signals showed a similar increase in vehicle (circles) and CC-292 (squares) treated nonloaded mice until day 28 (Figure 4). Between day 28 and day 33, BLI signals increased 2.7-fold in the vehicle treated and 1.8-fold in the CC-292 treated nonloaded mice (Figure 4). By day 33, vehicle and CC-292 treated nonloaded mice showed a similar degree of cortical destruction based on 3D renderings (Figure 2a,b, left panels). Changes in cortical thickness (Ct.Th) and cortical porosity (Ct.Po%) between day 13 and day 33 were not different between nonloaded vehicle treated and nonloaded CC-292 treated mice. Similarly, there was no change between day 13 to 33 measured in the microstructural parameters principal moments of inertia (I_min_, I_max_) cortical bone area (Ct.Ar), total cross-sectional area inside the periosteal envelope (Tt.Ar), cortical area fraction (Ct.Ar/Tt.Ar), cortical volumetric tissue mineral density (Ct.vTMD), and pore volume (Po.V) in metaphyseal cortical bone of nonloaded CC-292 treated mice compared to nonloaded vehicle treated mice (Supplementary Tables S1 and S2). In line with this, conventional histomorphometry analysis of the metaphyseal region of the cortical bone revealed no difference in bone formation parameters, such as mineralized surface area (MS/BS) or mineral apposition rate (MAR), at the endosteal or periosteal bone surface between nonloaded CC-292 treated and nonloaded vehicle treated mice (Figure 5a–d, Supplementary Table S3).

MicroCT analysis of the metaphyseal cancellous bone of day 33 compared to day 13 included the trabecular bone volume fraction (Tb.BV/TV, Figure 3c) and trabecular number (Tb.N, Figure 3d). Both parameters decreased in nonloaded vehicle and nonloaded CC-292 treated (Figure 3c,d) mice over time, indicating the effect of osteolytic trabecular bone loss due to MM injection. In the nonloaded mice, CC-292 treatment led to a greater decrease in the Tb.BV/TV compared to vehicle treatment (Figure 3c,d). Similarly, other trabecular parameters such as Tb.N revealed a more pronounced decrease in nonloaded mice after CC-292 treatment compared to vehicle treatment (Figure 3d, Supplementary Tables S4 and S5). Histomorphometric analysis did not show differences between CC-292 and vehicle treated nonloaded groups (Figure 5e,f, Supplementary Table S6).

MicroCT analysis of the diaphyseal cortical bone showed no changes in Ct.Th （Figure 3e) and Ct.Po% （Figure 3f) over time and between groups (Supplementary Tables S7 and S8). Histomorphometric analysis supported these findings (Figure 5g,h, Supplementary Table S9).

### 2.2. CC-292 Combined with Loading Prevents Bone Destruction but Is Not More Effective Than Loading Alone

Our previously published BLI data of the vehicle treated MM-injected mice showed that mechanical loading reduced tumor growth (see in [22] and Figure 4). In the CC-292 treated groups, BLI levels were not different between nonloaded and loaded mice (Figure 4). MicroCT 3D renderings showed a decrease in size and number of osteolytic cortical lesions over time (day 13 to day 33) in loaded compared to nonloaded mice from both vehicle and CC-292 treated groups (Figure 2a,b). MicroCT analysis of metaphyseal cortical bone revealed a similar increase in Ct.Th (Figure 3a) and a decrease in Ct.Po% (Figure 3b) in vehicle and CC-292 treated mice after loading from day 13 to day 33 (Supplementary Tables S1 and S2). Histomorphometric analysis revealed a slightly reduced MS/BS on the endosteal and periosteal surfaces in CC-292 treated loaded mice compared to vehicle treated loaded mice (Figure 5c, Supplementary Table S3). Loading did not change trabecular bone parameters such as Tb.BV/TV (Figure 3c) or Tb.N (Figure 3d) in the microCT analysis and MS/BS (Figure 5e) or MAR (Figure 5f) in the histomorphometric analysis (Supplementary Table S6). 

MicroCT analysis of the diaphyseal cortical bone demonstrated an increase in Ct.Th (Figure 3e) and Ct.Po% (Figure 3f) after loading in vehicle and CC-292 treated mice. Again, CC-292 treatment did not augment the effect of loading, as I_min_, I_max_, Ct.Ar, Tt.Ar, Ct.Ar./Tt.Ar., Ct.Th., Ct.vTMD, Ct.Po%, and Po.V were not significantly different between the CC-292 treated loaded mice and vehicle treated loaded mice (Figure 3e,f, Supplementary Tables S7 and S8).

Histomorphometric analysis showed a significant decrease in the MS/BS at the endosteal surface after loading in CC-292 compared to vehicle treated mice (Figure 5g, Supplementary Table S9). On the periosteal surface, loading led to an increase in the MS/BS area in both vehicle and CC-292 treated animals (Figure 5g), whereas MAR did not change (Figure 5h). Additional parameters of the microCT and histomorphometry analysis are given in the Supplementary Tables S1–S9.

## 3. Discussion

We examined whether CC-292 treatment alone or in combination with mechanical loading could maintain or even enhance bone mass and protect microstructure and formation, and thereby prevent bone destruction in the MOPC315.BM.Luc mouse model of MMBD [7,22,24]. To this end, we applied mechanical loading for three weeks and administered CC-292 five days a week during the loading period starting 14 days after intratibial MM cell inoculation.

MicroCT analysis of bones in CC-292 treated nonloaded mice revealed severe bone destruction in the metaphyseal cortical region after tumor cell inoculation during the study course. Microstructural parameters in the metaphyseal region of these mice showed decreased cortical thickness, increased cortical porosity, and decreased trabecular bone volume fraction and trabecular number, indicating the osteolytic activity of intratibially injected MOPC315.BM.Luc cells. In nonloaded mice, CC-292 treatment even led to further deterioration of the metaphyseal trabecular bone beyond the osteolytic effect of tumor cells when compared to vehicle treated nonloaded mice. During the 33 days after tumor cell injection, microstructural changes were minor in the mid-diaphyseal region of the tibiae. Our data provide evidence that CC-292 alone does not enhance bone formation, mass, or microstructure in the MOPC315.BM.Luc model. Eda et al. showed that CC-292 treatment in human MM.1S injected SCID mice reduced resorption in the lumbar vertebrae of mice resulting in improved trabecular microstructure [10]. In line with our observation, CC-292 treatment did not reduce tumor burden [10]. Treatment in SCID mice started immediately after tumor cell inoculation and continued for six weeks [10]. In our study, CC-292 treatment was applied for three weeks and started only after 14 days when tumor cells were already locally growing. Using the small-molecule inhibitor of BTK, LFM-A13, in a SCID-rab model with primary myeloma cells injected into rabbit bones, Bam and co-workers reported a suppressed osteoclast activity, less osteolytic resorption, and a moderate decrease in myeloma growth [31]. Again, the use of a different MMBD model and BTK inhibitor might account for the observed bone effects in this study [31].

We previously demonstrated the anabolic effect of mechanical loading in MOPC315.BM.Luc mice [22]. Similarly, mechanical loading prevented bone destruction and rescued bone mass in CC-292 treated mice. Our data and findings of Pagnotti and co-workers using low intensity vibration in a murine model of myeloma confirm the rationale for using physical stimuli as a countermeasure for osteolytic bone disease in MM [21,22]. In clinical settings, exercise training has already been proven to be feasible in patients suffering from MM [32,33]. We recently showed that whole-body vibration exercise benefits physical fitness and bone turnover in patients with monoclonal gammopathy of undetermined significance, a precursor condition of MMBD [29]. Based on our previous findings and the reports by Eda et al. that a combination treatment of CC-292 with carfilzomib improved bone mass [10], we further reasoned that the combination of mechanical loading with CC-292 enhances the osteoprotective effect of physical stimuli. However, we did not observe such outcomes in our study. CC-292 combined with loading prevented bone destruction, but combined treatment was no more effective in enhancing bone mass or microstructure than loading alone. This could be due to the more aggressive nature of the MOPC315.BM.Luc model compared to the MM.1S model used by Eda et al. [10]. As for histomorphometric analysis, the mineralization surface in CC-292 treated loaded mice was even reduced, indicating lower bone formation compared to vehicle treated mice.

While we did not observe the hypothesized additive effects of a combination of CC-292 with mechanical loading, other combinatory approaches with bone anabolic agents might prove beneficial. It was shown that transforming growth factor β (TGFβ) inhibition improves bone quality and fracture resistance in mice with human U266-GFP-luc myeloma [34]. Furthermore, pre-clinical studies have shown that anabolic pharmacological agents, such as sclerostin or DKK1 inhibitors, lead to increased bone mass and decreased osteolysis in mice with MM [15,16,17]. Combination with anti-resorptive agents, such as bisphosphonates, might be an option as well. However, there exist conflicting data on the effect of bisphosphonates on the adaptive response to loading, with Stadelman et al. [35] reporting negative interaction between zoledronate and loading and Feher et al. [36] showing no such interaction. Moreover, these studies used healthy animals.

This study is not without limitations. We used young 10-week-old mice in our study, while MM is prevalent in the ageing population [2,37]. The reduction principle of the 3Rs (replacement, reduction, and refinement) was implemented in the experiments, thus recently published vehicle treated mice were included for comparison [22]. Only female mice were used, as frequent fighting is observed in group-housed male mice, which can mask possible effects of mechanical loading [38]. In addition, we did not assess histological or serum biomarker levels of osteoclast activity. Lastly, the high genomic heterogeneity of the disease should be taken into consideration before extrapolating the results to other MM (sub)types [39].

In summary, key findings of our study are (1) CC-292 alone does not prevent bone destruction in the MOPC315.BM.Luc model, and (2) combinational therapy of CC-292 with mechanical loading is not more effective than mechanical loading alone in enhancing bone formation, mass, and microstructure in the presence of osteolytically active tumor cells after local inoculation. Future studies should consider combinatory approaches using physical stimuli with other anti-myeloma and anti-resorptive therapies for treatment of MMBD.

## 4. Materials and Methods

### 4.1. MOPC315.BM.Luc Cell Line and Reagents

The MOPC315.BM.Luc cell line was originally developed by Hofgaard et al. and then stably transfected with firefly luciferase, as previously described [23]. MOPC315.BM.Luc cells show tropism for bone marrow, are adapted to in vitro growth, and produce IgA protein (M315) as paraprotein. The MOPC315.BM.Luc cell line was maintained at 37 °C in a humidified 95% air, 5% CO_2_ atmosphere, and cultured in supplemented RPMI 1640 GlutaMax [22,23]. 

### 4.2. BLI

BLI was performed to confirm tumor cell inoculation and disease progression (Figure 4). Images were acquired 10 min after intraperitoneal injection of anesthetics (80 mg/mg ketamine, 16 mg/kg xylazine in PBS) mixed with luciferin (300 mg/kg), from ventral views. Imaging for whole-body tumor was performed on an IVIS Spectrum (University Hospital of Würzburg, Würzburg, Germany) or an IVIS Lumina (Charité, Universitätsmedizin Berlin, Berlin, Germany) system (both PerkinElmer, Waltham, MA, USA), and data were analyzed with Living Image 4.4 (PerkinElmer, Waltham, MA, USA) and Prism 7 software (GraphPad, San Diego, CA, USA).

### 4.3. Experimental Design

In the animal facility of the University Hospital Würzburg, seven-week-old, female BALB/c mice (*n* = 37, Charles River Laboratories, Göttingen, Germany) were received and acclimatized according to guidelines. Food and water access were provided ad libitum and mice were group housed (5 per cage). MOPC315.BM.Luc cells were intratibially inoculated in the left tibia as previously described (right limb served as internal control) [22], and mice were relocated to the animal facility of the Charité-Universitätsmedizin Berlin (Berlin, Germany). A short acclimatization period after transport followed. Using a simple randomization process, mice were divided into four groups: (1) vehicle treated nonloaded (*n* = 9), (2) vehicle treated loaded (*n* = 8), (3) CC-292 treated nonloaded (*n* = 6), and (4) CC-292 treated loaded (*n* = 8). Sample size was calculated based on power analysis. Mice were sacrificed at day 33 by cervical dislocation under anesthesia (60 mg/kg ketamine, 0.3 mg/kg medetomidine). During the experiment, five mice (one mouse from the nonloaded vehicle treated group, two mice from the loaded vehicle treated group, one mouse from the nonloaded CC-292 treated groups, and one mouse from the loaded CC-292 treated group) developed extramedullary disease and were sacrificed, while one mouse from the CC-292 treated nonloaded group died while under anesthesia during microCT. Data from these mice were not included in the final analysis. Local legal research animal welfare representatives approved the experiments, and all policies and procedures were followed (55.2-DMS-2532-2-3, Regierung von Unterfranken, Würzburg, G0027/15 LAGeSo Berlin). In order to implement the reduction principle of the 3Rs (replacement, reduction, and refinement), we compared our data from nonloaded and loaded mice treated with CC-292 with previously published data from loaded and nonloaded vehicle treated MM-injected mice [22].

### 4.4. Bruton’s Tyrosine Kinase Inhibitor CC-292

All 14 mice in the CC-292 treatment group were treated with the Bruton’s tyrosine kinase inhibitor CC-292 (Celgene Avilomics Research, Bedford, MA, USA). CC-292 and vehicle (1% CMC/0.1% Tween 80) were applied via oral gavage (30 mg/kg) 5 days a week (M-F) for three weeks starting on the first day of loading (Figure 1a). 

### 4.5. In Vivo Mechanical Loading

The 10-week-old BALB/c mice underwent in vivo cyclic compressive loading of the left tibia or were not loaded (Figure 1b). During the loading session, isoflurane (2% in 1.0 L/mic O_2_) was used for anesthesia of the mice. The loading followed a well-established protocol [40] and consisted of 216 loading cycles applied at 4 Hz, with a rest insertion of 5 s after 4 cycles (Figure 1c). Peak loads of −10 N were applied engendering 2000 µε at the medial surface of the tibial-midshaft as previously reported [22]. Loading commenced 14 days post MM injection and continued for 5 days/week (M-F) for 3 weeks. The right tibia served as an internal control in all animals. Mice which were not loaded were placed in the loading device to experience the same handling procedures.

### 4.6. In Vivo MicroCT Imaging

To assess changes in bone mass, mineral density, and microstructure, in vivo microCT (vivaCT 40, Scanco Medical, Brüttisellen, Switzerland) imaging was performed. Parameters for imaging were set as follows: isotropic voxel size of 10.5 µm, 55 kVp, 145 μA, 600 ms integration time, no frame averaging, and 180° rotation. Imaging was performed on day 13 post injection (1 day prior to loading), day 18, day 23, day 28, and day 33. Mice were anesthetized (60 mg/kg ketamine, 0.3 mg/kg medetomidine) and kept in a fixed position in a custom-made animal bed to mitigate motion artifacts. For both left and right tibia, a metaphyseal VOI as well as a diaphyseal VOI were defined. The metaphyseal VOI started at 105 µm below the most distal point of the growth plate, extended distally 10% of the total tibial length, and was chosen in the innate coordinate system of the scanner geometry. The diaphyseal VOI was set at the tibial midpoint and extended both distally and proximally 2.5% of the total tibia length. 

### 4.7. MicroCT Analysis

To reduce the effects of polychromatic X-ray absorption, a polynomial beam hardening correction is routinely applied in the reconstruction algorithm of the Scanco vivaCT 40. Using Otsu’s method [32], a global threshold of 688 mg hydroxyapatite (HA)/cm^3^ (cortical) or 467 mg HA/cm^3^ (trabecular) was used for separation of cortical and trabecular bone from background. As suggested [33], we measured the following cortical bone parameters: principal moments of inertia (I_max_, I_min_); cortical bone area = cortical volume/(number of slices⋅slice thickness) (Ct.Ar); total cross-sectional area inside the periosteal envelope (Tt.Ar); cortical area fraction (Ct.Ar/Tt.Ar); cortical thickness (Ct.Th); cortical volumetric tissue mineral density (Ct.vTMD); pore volume (Po.V); cortical porosity area, determined as the difference of the total cross-sectional area and the cortical bone area minus the medullary area (Ct.Po); and cortical porosity fraction (Ct.Po%). Cortical area and porosity include vascular porosity as well as osteolytic lesion area, but not lacunar porosity due to resolution constraints. Furthermore, trabecular bone parameters included bone volume fraction (BV/TV), trabecular thickness (Tb.Th), average number of trabeculae per unit length (Tb.N), trabecular separation (Tb.Sp), and trabecular volumetric tissue mineral density (Tb.vTMD) [33].

Finally, changes in the described parameters between the first scan (day 13) and the last scan (day 33) were calculated and assessed.

### 4.8. Dynamic Histomorphometry

Calcein was intra-peritoneally injected 12 and 3 days prior to euthanasia. Following dissection of both tibiae, bones were embedded in methyl methacrylate and calcein labels imaged and analyzed according to Parfitt et al. [34] and Foldes et al. [35]. Details can be found in supplemental material.

### 4.9. Statistical Analysis

To test for normality and homoscedasticity, the Shapiro–Wilk test and Folded F test for equal variances were performed. The between-subject effects of treatment (vehicle, CC-292) and loading (nonloaded mice, loaded mice) and the within-subject effect of limb (right nonloaded control limb, left loaded limb), as well as interactions between these terms, were assessed using a repeated measures ANOVA (SAS 9.3, Cary, NC, USA). Differences between treatment and loading condition were assessed by either paired or unpaired t-tests as applicable or a Tukey–Kramer post-hoc test. Unless otherwise indicated, all results reported were significant, * *p* < 0.05 and are presented as mean ± standard deviation.

## Data Availability

Data are contained within the article or supplementary material.

## Supplementary Materials

The following are available online at https://www.mdpi.com/article/10.3390/ijms22083840/s1, Materials and Methods: Dynamic histomorphometry, Table S1: MicroCT parameters in metaphyseal cortical bone, Table S2: Changes in microCT parameters in metaphyseal cortical bone, Table S3: Dynamic cortical bone formation indices in the metaphysis, Table S4: MicroCT parameters in trabecular bone compartment, Table S5: Changes in microCT parameters in trabecular bone compartment, Table S6: Dynamic cancellous bone formation indices in the metaphysis, Table S7: MicroCT parameters in diaphyseal cortical bone, Table S8: Changes in microCT parameters in diaphyseal cortical bone, Table S9: Dynamic cortical bone formation indices in the diaphysis.
