# Supplementary material for: Prevention of Bone Destruction by Mechanical Loading Is Not Enhanced by the Bruton’s Tyrosine Kinase Inhibitor CC-292 in Myeloma Bone Disease
Source: Int J Mol Sci. 2021 Apr 7;22(8):3840. doi: 10.3390/ijms22083840 (PMC8067978; doi:10.3390/ijms22083840)
Supplement: Supplementary file 1 [file ijms-22-03840-s001.pdf]

Prevention of bone destruction by mechanical loading is not enhanced by the Bruton´s tyrosine kinase inhibitor CC-292 in myeloma bone disease

Fani Ziouti*, Maximilian Rummler*, Beatrice Steyn, Tobias Thiele, Anne Seliger, Georg N. Duda, Bjarne Bogen, Bettina M. Willie*, and Franziska Jundt*
* authors contributed equally

Supplementary material

Materials and Methods

Dynamic histomorphometry

Calcein (30 mg/kg) was administered via intraperitoneal injection 12 and 3 days prior to euthanasia. Following dissection of the left and the right tibiae, bones were fixed in 70% ethanol and dehydrated in ascending grades of ethanol up to absolute. Bones were then cleaned in xylene and embedded in polymethyl methacrylate. Samples were sectioned in the sagittal plane at the proximal tibia to analyze trabecular and cortical bone and in the transverse plane at the mid-shaft to analyze cortical bone in similar regions. Sections were positioned on microscopy slides and viewed at a magnification of 20x using a laser scanning confocal microscope (Zeiss LSM780, Jena, Germany) for evidence of fluorochrome labels. Images were analyzed using a commercial histomorphometric system (BIOQUANT OSTEO, Nashville, TN, USA). For trabecular and cortical bone, the single- and double-labeled surface per bone surface (sLS/BS, dLS/BS), mineralizing surface per bone surface (MS/BS), mineral apposition rate (MAR), and bone-formation rate (BFR/BS), were analyzed as recommended [37]. MS/BS was calculated as 0.5x sLS/BS + dLS/BS. When a specimen had no double-labeled surface (dLS/BS = 0), it did not contribute for MAR and BFR/BS [38]. For trabecular bone, MAR was determined by measuring 5 to 10 double labels within one section per tibia and five measurements along the span of each double label. The thickness of newly mineralized bone at the surface of the trabeculae was averaged along the active bone-forming surface, divided by the 9-day labeling interval, and

expressed as the MAR in units of micrometer per day. For cortical bone, the entire endocortical (Ec) and periosteal (Ps) surfaces in both regions were analyzed.

Results

	vehicle treated				CC-292 treated			
	nonloaded		loaded		nonloaded		loaded	
	n = 9		n = 8		n = 6		n = 8	
Day 13	right limb	left limb	right limb	left limb	right limb	left limb	right limb	left limb
$I_{min}^{a,c}$ [mm^4]	0.27 ± 0.03	0.29 ± 0.03	0.28 ± 0.03	0.31 ± 0.03	0.27 ± 0.02	0.28 ± 0.01	0.24 ± 0.02	0.26 ± 0.04
$I_{max}^{a,c}$ [mm^4]	0.40 ± 0.05	0.46 ± 0.07	0.40 ± 0.05	0.49 ± 0.07	0.42 ± 0.05	0.42 ± 0.02	0.34 ± 0.04	0.38 ± 0.06
Ct.Ar^c [mm^2]	0.89 ± 0.05	0.98 ± 0.06	0.92 ± 0.06	1.04 ± 0.06	0.95 ± 0.11	1.00 ± 0.08	0.85 ± 0.05	0.95 ± 0.05
Tt.Ar^c [mm^2]	1.11 ± 0.06	1.21 ± 0.07	1.09 ± 0.08	1.24 ± 0.09	1.15 ± 0.16	1.18 ± 0.11	1.03 ± 0.08	1.13 ± 0.08
Ct.Ar/Tt.Ar^c [mm^2/mm^2]	0.80 ± 0.01	0.81 ± 0.01	0.85 ± 0.02	0.84 ± 0.03	0.83 ± 0.03	0.85 ± 0.02	0.82 ± 0.04	0.84 ± 0.03
Ct.Th^c [mm]	0.13 ± 0.00	0.14 ± 0.00	0.13 ± 0.01	0.14 ± 0.01	0.14 ± 0.02	0.15 ± 0.02	0.13 ± 0.01	0.14 ± 0.01
Ct.vTMD^{a,c} [mgHA/cm^3]	1088 ± 15	1079 ± 12	1106 ± 16	1097 ± 19	1159 ± 51	1154 ± 51	1153 ± 43	1148 ± 43
Ct.Po%^{c} [%]	25 ± 2	24 ± 1	18 ± 3	19 ± 4	20 ± 4	18 ± 3	22 ± 6	19 ± 5
Po.V [mm^3]	0.36 ± 0.04	0.38 ± 0.02	0.26 ± 0.05	0.32 ± 0.07	0.32 ± 0.08	0.29 ± 0.06	0.31 ± 0.08	0.29 ± 0.08
Day 18								
I_{min} [mm^4]	0.28 ± 0.04	0.31 ± 0.03	0.29 ± 0.02	0.35 ± 0.03	0.28 ± 0.02	0.28 ± 0.01	0.24 ± 0.02	0.29 ± 0.05
I_{max} [mm^4]	0.40 ± 0.05	0.45 ± 0.07	0.39 ± 0.03	0.54 ± 0.08	0.41 ± 0.06	0.43 ± 0.02	0.34 ± 0.03	0.42 ± 0.08
Ct.Ar [mm^2]	0.91 ± 0.05	0.97 ± 0.07	0.95 ± 0.04	1.18 ± 0.04	0.96 ± 0.12	1.02 ± 0.08	0.87 ± 0.05	1.05 ± 0.07
Tt.Ar [mm^2]	1.10 ± 0.07	1.18 ± 0.10	1.10 ± 0.05	1.35 ± 0.04	1.12 ± 0.16	1.19 ± 0.12	1.04 ± 0.09	1.22 ± 0.08
Ct.Ar/Tt.Ar [mm^2/mm^2]	0.83 ± 0.01	0.82 ± 0.02	0.86 ± 0.02	0.88 ± 0.01	0.86 ± 0.03	0.86 ± 0.03	0.84 ± 0.04	0.86 ± 0.04
Ct.Th [mm]	0.13 ± 0.00	0.14 ± 0.01	0.14 ± 0.01	0.17 ± 0.01	0.14 ± 0.02	0.15 ± 0.02	0.13 ± 0.01	0.16 ± 0.01
Ct.vTMD [mgHA/cm^3]	1102 ± 20	1097 ± 17	1122 ± 16	1133 ± 15	1178 ± 42	1176 ± 41	1174 ± 40	1180 ± 31
Ct.Po% [%]	20 ± 2	22 ± 3	16 ± 3	14 ± 1	17 ± 4	16 ± 4	20 ± 5	16 ± 5
Po.V [mm^3]	0.27 ± 0.11	0.31 ± 0.13	0.25 ± 0.04	0.27 ± 0.02	0.26 ± 0.09	0.27 ± 0.08	0.28 ± 0.08	0.27 ± 0.08
Day 23								
I_{min} [mm^4]	0.29 ± 0.03	0.32 ± 0.03	0.31 ± 0.03	0.41 ± 0.04	0.29 ± 0.03	0.30 ± 0.02	0.25 ± 0.02	0.31 ± 0.05
I_{max} [mm^4]	0.42 ± 0.05	0.46 ± 0.06	0.41 ± 0.03	0.61 ± 0.09	0.44 ± 0.07	0.44 ± 0.03	0.34 ± 0.03	0.48 ± 0.09
Ct.Ar [mm^2]	0.99 ± 0.06	1.00 ± 0.05	1.03 ± 0.05	1.35 ± 0.08	1.05 ± 0.12	1.06 ± 0.08	0.90 ± 0.07	1.19 ± 0.10
Tt.Ar [mm^2]	1.16 ± 0.07	1.22 ± 0.07	1.18 ± 0.06	1.54 ± 0.08	1.23 ± 0.18	1.26 ± 0.16	1.07 ± 0.12	1.37 ± 0.11
Ct.Ar/Tt.Ar [mm^2/mm^2]	0.85 ± 0.01	0.82 ± 0.01	0.87 ± 0.01	0.88 ± 0.01	0.85 ± 0.04	0.85 ± 0.05	0.85 ± 0.05	0.86 ± 0.04
Ct.Th [mm]	0.15 ± 0.01	0.14 ± 0.00	0.16 ± 0.01	0.19 ± 0.01	0.16 ± 0.02	0.16 ± 0.01	0.14 ± 0.01	0.18 ± 0.01
Ct.vTMD [mgHA/cm^3]	1115 ± 17	1113 ± 15	1130 ± 17	1162 ± 12	1189 ± 39	1175 ± 30	1189 ± 33	1205 ± 15
Ct.Po% [%]	18 ± 2	22 ± 2	15 ± 1	14 ± 2	17 ± 6	18 ± 6	18 ± 7	16 ± 5
Po.V [mm^3]	0.28 ± 0.03	0.35 ± 0.04	0.25 ± 0.02	0.31 ± 0.04	0.29 ± 0.11	0.32 ± 0.12	0.27 ± 0.11	0.30 ± 0.09
Day 28								
I_{min} [mm^4]	0.30 ± 0.03	0.32 ± 0.03	0.32 ± 0.03	0.43 ± 0.05	0.30 ± 0.03	0.30 ± 0.01	0.25 ± 0.02	0.32 ± 0.05
I_{max} [mm^4]	0.42 ± 0.05	0.44 ± 0.05	0.42 ± 0.04	0.64 ± 0.10	0.44 ± 0.08	0.44 ± 0.03	0.34 ± 0.03	0.49 ± 0.08
Ct.Ar [mm^2]	1.02 ± 0.07	0.98 ± 0.04	1.07 ± 0.06	1.38 ± 0.10	1.08 ± 0.13	1.06 ± 0.05	0.94 ± 0.06	1.22 ± 0.08
Tt.Ar [mm^2]	1.19 ± 0.07	1.21 ± 0.06	1.21 ± 0.06	1.56 ± 0.09	1.26 ± 0.19	1.26 ± 0.12	1.11 ± 0.10	1.40 ± 0.09
Ct.Ar/Tt.Ar [mm^2/mm^2]	0.86 ± 0.01	0.81 ± 0.02	0.89 ± 0.01	0.89 ± 0.02	0.87 ± 0.04	0.84 ± 0.05	0.85 ± 0.05	0.87 ± 0.04
Ct.Th [mm]	0.16 ± 0.01	0.14 ± 0.00	0.17 ± 0.01	0.20 ± 0.02	0.17 ± 0.02	0.16 ± 0.01	0.15 ± 0.01	0.19 ± 0.01
Ct.vTMD [mgHA/cm^3]	1142 ± 11	1128 ± 14	1153 ± 12	1183 ± 13	1206 ± 32	1192 ± 24	1201 ± 32	1220 ± 14
Ct.Po% [%]	16 ± 1	23 ± 3	13 ± 1	13 ± 2	16 ± 6	19 ± 7	18 ± 7	15 ± 5
Po.V [mm^3]	0.27 ± 0.01	0.37 ± 0.05	0.22 ± 0.01	0.28 ± 0.04	0.29 ± 0.12	0.33 ± 0.13	0.28 ± 0.11	0.30 ± 0.10
Day 33								
$I_{min}^{a,b,c}$ [mm^4]	0.30 ± 0.04	0.31 ± 0.03	0.33 ± 0.04	0.43 ± 0.05	0.29 ± 0.03	0.30 ± 0.02	0.26 ± 0.02	0.32 ± 0.05
$I_{max}^{a,b,c}$[mm^4]	0.42 ± 0.05	0.42 ± 0.04	0.43 ± 0.05	0.62 ± 0.08	0.42 ± 0.07	0.40 ± 0.03	0.33 ± 0.03	0.48 ± 0.07
Ct.Ar^{a,b,c} [mm^2]	1.04 ± 0.07	0.95 ± 0.03	1.10 ± 0.07	1.38 ± 0.10	1.08 ± 0.13	1.02 ± 0.04	0.95 ± 0.05	1.21 ± 0.09
Tt.Ar^{b,c} [mm^2]	1.20 ± 0.08	1.18 ± 0.06	1.25 ± 0.06	1.57 ± 0.10	1.29 ± 0.19	1.27 ± 0.09	1.11 ± 0.09	1.40 ± 0.10
Ct.Ar/Tt.Ar^{b,c} [mm^2/mm^2]	0.87 ± 0.01	0.80 ± 0.02	0.88 ± 0.01	0.88 ± 0.02	0.84 ± 0.05	0.81 ± 0.04	0.85 ± 0.05	0.86 ± 0.04
Ct.Th^{b,c} [mm]	0.16 ± 0.01	0.14 ± 0.01	0.17 ± 0.01	0.20 ± 0.02	0.17 ± 0.02	0.15 ± 0.01	0.16 ± 0.01	0.19 ± 0.01
Ct.vTMD^{a,b} [mgHA/mm^3]	1157 ± 14	1137 ± 15	1179 ± 11	1198 ± 15	1215 ± 29	1207 ± 34	1217 ± 32	1231 ± 14
Ct.Po%^{b,c} [%]	15 ± 1	24 ± 4	14 ± 2	14 ± 3	19 ± 7	24 ± 6	17 ± 6	16 ± 6
Po.V^{b,c} [mm^3]	0.26 ± 0.02	0.37 ± 0.06	0.25 ± 0.02	0.32 ± 0.05	0.35 ± 0.15	0.41 ± 0.11	0.27 ± 0.10	0.31 ± 0.10

Table S1: **MicroCT parameters in metaphyseal cortical bone,** the table shows cortical bone parameters in the metaphysis of both limbs on five different experimental days in all experimental groups. Parameters are shown as mean ± standard deviation. *ANOVA main effects:* (a) treatment, (b) loading, (c) limb, post-hoc tests not shown.

	vehicle treated				CC-292 treated			
	nonloaded		loaded		nonloaded		loaded	
	n = 9		n = 8		n = 6		n = 8	
Day 33-13	right limb	left limb	right limb	left limb	right limb	left limb	right limb	left limb
$\Delta I_{min}^{a,b,c}$ [mm^4]	0.03 ± 0.02	0.02 ± 0.01	0.04 ± 0.01	0.12 ± 0.04	0.02 ± 0.01	0.02 ± 0.03	0.02 ± 0.02	0.07 ± 0.02
$\Delta I_{max}^{b,c}$ [mm^4]	0.02 ± 0.04	-0.03 ± 0.05	0.03 ± 0.02	0.13 ± 0.05	0.00 ± 0.03	-0.02 ± 0.03	-0.01 ± 0.03	0.10 ± 0.05
ΔCt.Ar^{a,b} [mm^2]	0.15 ± 0.05	-0.03 ± 0.04	0.18 ± 0.03	0.34 ± 0.10	0.13 ± 0.03	0.02 ± 0.07	0.10 ± 0.04	0.26 ± 0.07
ΔTt.Ar^{b,c} [mm^2]	0.08 ± 0.05	-0.04 ± 0.04	0.16 ± 0.04	0.33 ± 0.10	0.14 ± 0.04	0.08 ± 0.09	0.08 ± 0.04	0.27 ± 0.06
ΔCt.Ar/Tt.Ar^{a,b,c} [mm^2/mm^2]	0.07 ± 0.02	0.00 ± 0.03	0.03 ± 0.03	0.03 ± 0.03	0.01 ± 0.03	-0.04 ± 0.04	0.03 ± 0.02	0.02 ± 0.02
ΔCt.Th^{b} [mm]	0.03 ± 0.01	0.00 ± 0.01	0.04 ± 0.01	0.06 ± 0.02	0.03 ± 0.01	0.00 ± 0.01	0.03 ± 0.01	0.05 ± 0.01
ΔCt.vTMD^{b,c} [$mgHA/cm^3$]	69 ± 11	57 ± 10	73 ± 7	100 ± 10	56 ± 27	53 ± 36	64 ± 18	83 ± 32
ΔCt.Po%^{a,b,c} [%]	-10 ± 3	0 ± 4	-4 ± 4	-5 ± 4	-1 ± 4	6 ± 5	-5 ± 2	-3 ± 3
ΔPo.V^{a,c} [mm^3]	-0.10 ± 0.04	0.00 ± 0.06	-0.02 ± 0.06	0.00 ± 0.07	0.03 ± 0.08	0.12 ± 0.08	-0.03 ± 0.04	0.02 ± 0.06

Table S2: **Changes in microCT parameters in metaphyseal cortical bone,** the table shows the changes in cortical bone parameters in the metaphysis of both limbs between day 13 and day 33 in all experimental groups. Parameters are shown as mean ± standard deviation. *ANOVA main effects:* (a) treatment, (b) loading, (c) limb, post-hoc tests not shown.

		vehicle treated				CC-292 treated			
		nonloaded		loaded		nonloaded		loaded	
		n = 9		n = 8		n = 6		n = 8	
		right limb	left limb	right limb	left limb	right limb	left limb	right limb	left limb
Ec.sLS/BS^{a}	%	0.6 ± 0.2	0.5 ± 0.1	0.5 ± 0.1	0.6 ± 0.3	0.2 ± 0.2	0.3 ± 0.2	0.1 ± 0.1	0.3 ± 0.1
Ec.dLS/BS	%	0.2 ± 0.1	0.2 ± 0.1	0.2 ± 0.1	0.3 ± 0.2	0.3 ± 0.1	0.1 ± 0.1	0.2 ± 0.1	0.1 ± 0.1
Ec.MS/BS^{a}	%	0.4 ± 0.1	0.4 ± 0.1	0.4 ± 0.1	0.5 ± 0.2	0.3 ± 0.1	0.3 ± 0.1	0.3 ± 0.1	0.3 ± 0.1
Ec.MAR(9d)	(µm/day)	2.6 ± 0.5	2.6 ± 0.8	2.1 ± 0.6	3.1 ± 1.8	3.6 ± 1.7	3.6 ± 2.4	3.4 ± 0.8	2.0 ± 1.1
Ec.BFR^{a}	(µm/day)	1.4 ± 0.3	1.1 ± 0.6	0.8 ± 0.2	1.8 ± 1.2	1.1 ± 0.2	0.9 ± 0.5	1.0 ± 0.3	0.5 ± 0.2
Ps.sLS/BS^{a,c}	%	0.8 ± 0.2	0.4 ± 0.2	0.7 ± 0.2	0.7 ± 0.3	0.5 ± 0.4	0.4 ± 0.1	0.3 ± 0.1	0.2 ± 0.1
Ps.dLS/BS^{b}	%	0.1 ± 0.1	0.2 ± 0.1	0.2 ± 0.1	0.3 ± 0.3	0.2 ± 0.2	0.1 ± .	0.2 ± 0.1	0.3 ± 0.1
Ps.MS/BS^{a,b}	%	0.4 ± 0.1	0.3 ± 0.1	0.5 ± 0.2	0.6 ± 0.2	0.4 ± 0.2	0.2 ± 0.1	0.3 ± 0.1	0.4 ± 0.1
Ps.MAR(9d)^{b,c}	(µm/day)	1.7 ± 0.7	1.4 ± 1.0	1.8 ± 0.5	3.0 ± 0.8	1.4 ± 0.3	1.5 ± .	1.6 ± 1.0	3.9 ± 2.8
Ps.BFR^{a,b}	(µm/day)	0.7 ± 0.3	0.5 ± 0.5	1.1 ± 0.5	1.5 ± 0.5	0.5 ± 0.2	0.5 ± .	0.6 ± 0.3	1.4 ± 0.9

Table S3: **Dynamic cortical bone formation indices in the metaphysis** from mice either nonloaded or loaded and treated with vehicle or CC-292. Parameters are shown as mean ± standard deviation. *ANOVA main effects:* (a) treatment, (b) loading, (c) limb, post-hoc tests not shown.

	vehicle treated				CC-292 treated			
	nonloaded		loaded		nonloaded		loaded	
	n = 9		n = 8		n = 6		n = 8	
Day 13	right limb	left limb	right limb	left limb	right limb	left limb	right limb	left limb
**Tb.BV/TV[a,c] [mm^3/mm^3]**	0.11 ± 0.02	0.15 ± 0.02	0.14 ± 0.01	0.19 ± 0.05	0.15 ± 0.03	0.24 ± 0.02	0.15 ± 0.05	0.25 ± 0.07
**Tb.Th[a,c] [mm]**	0.06 ± 0.00	0.07 ± 0.00	0.06 ± 0.00	0.07 ± 0.01	0.06 ± 0.00	0.08 ± 0.01	0.06 ± 0.00	0.08 ± 0.01
**Tb.N[a,b] [1/mm]**	2.51 ± 0.34	2.41 ± 0.23	2.99 ± 0.30	3.08 ± 0.61	3.18 ± 0.34	3.24 ± 0.19	3.08 ± 0.52	3.26 ± 0.50
**Tb.Sp[a,b] [mm]**	0.42 ± 0.06	0.45 ± 0.04	0.35 ± 0.04	0.37 ± 0.08	0.33 ± 0.04	0.35 ± 0.03	0.34 ± 0.05	0.36 ± 0.06
**Tb.vTMD[a,c] [mgHA/cm^3]**	835 ± 22	817 ± 18	864 ± 13	849 ± 16	916 ± 45	899 ± 27	927 ± 42	887 ± 33
Day 18								
**Tb.BV/Tv [mm^3/mm^3]**	0.13 ± 0.02	0.14 ± 0.02	0.16 ± 0.02	0.18 ± 0.03	0.17 ± 0.04	0.19 ± 0.04	0.16 ± 0.05	0.22 ± 0.05
**Tb.Th [mm]**	0.06 ± 0.00	0.07 ± 0.00	0.06 ± 0.00	0.07 ± 0.01	0.47 ± 0.98	0.53 ± 1.12	0.06 ± 0.01	0.07 ± 0.01
**Tb.N [1/mm]**	2.68 ± 0.33	2.20 ± 0.33	3.26 ± 0.15	2.77 ± 0.46	3.08 ± 0.66	2.68 ± 0.66	3.05 ± 0.49	3.00 ± 0.36
**Tb.Sp [mm]**	0.40 ± 0.05	0.49 ± 0.07	0.32 ± 0.02	0.39 ± 0.07	0.27 ± 0.11	0.32 ± 0.13	0.35 ± 0.05	0.37 ± 0.06
**Tb.vTMD [mgHA/cm^3]**	851 ± 30	854 ± 22	872 ± 7	882 ± 16	956 ± 56	949 ± 23	950 ± 25	943 ± 31
Day 23								
**Tb.BV/Tv [mm^3/mm^3]**	0.12 ± 0.01	0.09 ± 0.01	0.14 ± 0.01	0.14 ± 0.03	0.15 ± 0.06	0.14 ± 0.05	0.14 ± 0.05	0.16 ± 0.05
**Tb.Th [mm]**	0.06 ± 0.00	0.06 ± 0.00	0.06 ± 0.00	0.07 ± 0.01	0.07 ± 0.01	0.06 ± 0.00	0.06 ± 0.00	0.07 ± 0.01
**Tb.N [1/mm]**	2.67 ± 0.27	1.82 ± 0.30	3.22 ± 0.16	2.10 ± 0.54	3.06 ± 0.43	2.27 ± 0.30	2.95 ± 0.40	2.31 ± 0.30
**Tb.Sp [mm]**	0.39 ± 0.05	0.59 ± 0.08	0.32 ± 0.02	0.53 ± 0.11	0.35 ± 0.05	0.48 ± 0.06	0.36 ± 0.05	0.47 ± 0.07
**Tb.vTMD [mgHA/cm^3]**	876 ± 10	890 ± 17	890 ± 13	932 ± 18	923 ± 58	921 ± 53	961 ± 21	991 ± 23
Day 28								
**Tb.BV/Tv [mm^3/mm^3]**	0.10 ± 0.01	0.07 ± 0.02	0.14 ± 0.01	0.12 ± 0.03	0.13 ± 0.05	0.09 ± 0.05	0.12 ± 0.05	0.12 ± 0.05
**Tb.Th [mm]**	0.06 ± 0.00	0.05 ± 0.00	0.06 ± 0.00	0.07 ± 0.01	0.06 ± 0.00	0.05 ± 0.00	0.06 ± 0.00	0.07 ± 0.01
**Tb.N [1/mm]**	2.30 ± 0.40	1.53 ± 0.17	3.04 ± 0.23	1.81 ± 0.54	2.76 ± 0.37	1.74 ± 0.07	2.68 ± 0.40	1.73 ± 0.12
**Tb.Sp [mm]**	0.46 ± 0.08	0.67 ± 0.06	0.34 ± 0.03	0.62 ± 0.13	0.38 ± 0.06	0.60 ± 0.03	0.40 ± 0.06	0.61 ± 0.05
**Tb.vTMD [mgHA/cm^3]**	887 ± 13	915 ± 21	905 ± 10	961 ± 24	964 ± 33	945 ± 55	962 ± 30	1016 ± 20
Day 33								
**Tb.BV/TV[c] [mm^3/mm^3]**	0.10 ± 0.01	0.06 ± 0.01	0.12 ± 0.02	0.10 ± 0.03	0.12 ± 0.04	0.06 ± 0.03	0.10 ± 0.03	0.10 ± 0.05
**Tb.Th [mm]**	0.05 ± 0.00	0.05 ± 0.01	0.06 ± 0.00	0.07 ± 0.01	0.06 ± 0.01	0.05 ± 0.00	0.26 ± 0.58	0.32 ± 0.71
**Tb.N[c] [1/mm]**	1.96 ± 0.33	1.49 ± 0.14	2.63 ± 0.38	1.73 ± 0.49	2.38 ± 0.40	1.56 ± 0.11	2.08 ± 0.50	1.54 ± 0.20
**Tb.Sp[b,c] [mm]**	0.54 ± 0.09	0.70 ± 0.05	0.40 ± 0.06	0.64 ± 0.13	0.45 ± 0.07	0.66 ± 0.05	0.42 ± 0.15	0.60 ± 0.23
**Tb.vTMD[b,c] [mgHA/cm^3]**	895 ± 21	908 ± 22	927 ± 16	989 ± 28	897 ± 64	878 ± 103	986 ± 33	1038 ± 43

Table S4: **MicroCT parameters in trabecular bone compartment,** the table shows trabecular bone parameters in the metaphysis of both limbs on five different experimental days in all experimental groups. Parameters are shown as mean ± standard deviation. *ANOVA main effects:* (a) treatment, (b) loading, (c) limb, post-hoc tests not shown.

	vehicle treated				CC-292 treated			
	nonloaded		loaded		nonloaded		loaded	
	n = 9		n = 8		n = 6		n = 8	
Day 33-13	right limb	left limb	right limb	left limb	right limb	left limb	right limb	left limb
**ΔTb.BV/TV[a,c] [mm^3/mm^3]**	-0.01 ± 0.03	-0.09 ± 0.02	-0.02 ± 0.02	-0.09 ± 0.05	-0.03 ± 0.03	-0.17 ± 0.04	-0.05 ± 0.03	-0.15 ± 0.06
**ΔTb.Th [mm]**	0.00 ± 0.01	-0.02 ± 0.01	0.00 ± 0.00	0.00 ± 0.01	0.00 ± 0.01	-0.03 ± 0.01	0.20 ± 0.58	0.24 ± 0.71
**ΔTb.N[a,c] [1/mm]**	-0.56 ± 0.43	-0.92 ± 0.19	-0.36 ± 0.33	-1.35 ± 0.61	-0.80 ± 0.39	-1.68 ± 0.21	-1.00 ± 0.87	-1.72 ± 0.46
**ΔTb.Sp[c] [mm]**	0.13 ± 0.09	0.24 ± 0.04	0.05 ± 0.05	0.27 ± 0.13	0.12 ± 0.06	0.31 ± 0.06	0.07 ± 0.13	0.24 ± 0.23
**ΔTb.vTMD[a,b,c] [mgHA/cm^3]**	60 ± 21	91 ± 32	63 ± 16	140 ± 25	-19 ± 68	-20 ± 112	59 ± 29	151 ± 50

Table S5: **Changes in microCT parameters in trabecular bone compartment,** the table shows the changes in trabecular bone parameters in the metaphysis of both limbs between day 13 and day 33 in all experimental groups. Parameters are shown as mean ± standard deviation. *ANOVA main effects:* (a) treatment, (b) loading, (c) limb, post-hoc tests not shown.

		vehicle treated				CC-292 treated			
		nonloaded n = 9		loaded n = 8		nonloaded n = 6		loaded n = 8	
		right limb	left limb	right limb	left limb	right limb	left limb	right limb	left limb
**Tb.sLS/BS**	**%**	0.3 ± 0.1	0.3 ± 0.1	0.3 ± 0.3	0.3 ± 0.0	0.3 ± 0.3	0.4 ± 0.2	0.3 ± 0.1	0.4 ± 0.2
**Tb.dLS/BS**	**%**	0.1 ± 0.0	0.1 ± 0.0	0.2 ± 0.1	0.1 ± 0.1	0.2 ± 0.1	0.1 ± 0.1	0.1 ± 0.1	0.1 ± 0.0
**Tb.MS/BS**	**%**	0.2 ± 0.1	0.2 ± 0.1	0.2 ± 0.1	0.2 ± 0.1	0.2 ± 0.1	0.2 ± 0.1	0.2 ± 0.1	0.3 ± 0.1
**Tb.MAR(9d)**	**(µm/day)**	1.8 ± 0.3	1.3 ± 0.5	2.0 ± 0.4	2.4 ± 1.3	2.0 ± 0.4	2.4 ± 1.0	2.5 ± 1.6	3.3 ± 1.7
**Tb.BFR**	**(µm/day)**	0.4 ± 0.2	0.3 ± 0.1	0.5 ± 0.2	0.6 ± 0.3	0.5 ± 0.2	0.6 ± 0.3	0.8 ± 0.7	0.9 ± 0.6

Table S6: **Dynamic cancellous bone formation indices in the metaphysis** from mice either nonloaded or loaded and treated with vehicle or CC-292. Parameters are shown as mean ± standard deviation. *ANOVA main effects:* (a) treatment, (b) loading, (c) limb, post-hoc tests not shown.

	vehicle treated				CC-292 treated			
	nonloaded n = 9		loaded n = 8		nonloaded n=6		loaded n=8	
Day 13	right limb	left limb	right limb	left limb	right limb	left limb	right limb	left limb
I_{min} [mm^4]	0.04 ± 0.00	0.04 ± 0.00	0.04 ± 0.01	0.05 ± 0.00	0.04 ± 0.00	0.05 ± 0.01	0.04 ± 0.00	0.04 ± 0.00
I_{max} [mm^4]	0.06 ± 0.00	0.06 ± 0.01	0.07 ± 0.01	0.06 ± 0.01	0.06 ± 0.01	0.06 ± 0.01	0.06 ± 0.00	0.06 ± 0.01
Ct.Ar [mm^2]	0.55 ± 0.02	0.55 ± 0.02	0.58 ± 0.03	0.58 ± 0.03	0.58 ± 0.02	0.57 ± 0.03	0.55 ± 0.02	0.58 ± 0.03
Tt.Ar[a,c] [mm^2]	0.60 ± 0.02	0.59 ± 0.02	0.62 ± 0.03	0.63 ± 0.02	0.64 ± 0.04	0.64 ± 0.05	0.62 ± 0.04	0.66 ± 0.04
Ct.Ar/Tt.Ar [mm^2/mm^2]	0.92 ± 0.01	0.92 ± 0.00	0.92 ± 0.00	0.92 ± 0.01	0.90 ± 0.03	0.90 ± 0.03	0.89 ± 0.03	0.89 ± 0.03
Ct.Th [mm]	0.20 ± 0.01	0.20 ± 0.01	0.21 ± 0.01	0.21 ± 0.01	0.21 ± 0.01	0.21 ± 0.01	0.20 ± 0.01	0.21 ± 0.01
Ct.vTMD[a] [mgHA/cm^3]	1300 ± 11	1313 ± 11	1305 ± 7	1309 ± 19	1353 ± 26	1349 ± 29	1347 ± 25	1344 ± 31
Ct.Po%[a] [%]	9 ± 1	8 ± 0	9 ± 0	9 ± 1	12 ± 3	11 ± 4	13 ± 4	13 ± 4
Po.V[a] [mm^3]	0.04 ± 0.00	0.04 ± 0.00	0.04 ± 0.00	0.04 ± 0.00	0.05 ± 0.02	0.05 ± 0.02	0.06 ± 0.02	0.06 ± 0.02
Day 18								
I_{min} [mm^4]	0.04 ± 0.00	0.04 ± 0.00	0.05 ± 0.00	0.05 ± 0.00	0.05 ± 0.00	0.05 ± 0.00	0.04 ± 0.00	0.04 ± 0.00
I_{max} [mm^4]	0.06 ± 0.01	0.06 ± 0.01	0.07 ± 0.01	0.07 ± 0.01	0.07 ± 0.00	0.07 ± 0.01	0.06 ± 0.00	0.06 ± 0.01
Ct.Ar [mm^2]	0.56 ± 0.02	0.56 ± 0.02	0.59 ± 0.03	0.61 ± 0.02	0.58 ± 0.02	0.58 ± 0.02	0.56 ± 0.02	0.61 ± 0.03
Tt.Ar [mm^2]	0.61 ± 0.02	0.60 ± 0.03	0.64 ± 0.03	0.66 ± 0.02	0.65 ± 0.04	0.66 ± 0.04	0.63 ± 0.04	0.68 ± 0.03
Ct.Ar/Tt.Ar [mm^2/mm^2]	0.92 ± 0.00	0.93 ± 0.00	0.92 ± 0.00	0.93 ± 0.00	0.89 ± 0.03	0.89 ± 0.03	0.89 ± 0.03	0.90 ± 0.03
Ct.Th [mm]	0.20 ± 0.01	0.21 ± 0.01	0.21 ± 0.01	0.22 ± 0.01	0.21 ± 0.01	0.21 ± 0.01	0.21 ± 0.01	0.23 ± 0.01
Ct.vTMD [mgHA/cm^3]	1307 ± 10	1321 ± 14	1316 ± 5	1320 ± 9	1359 ± 23	1345 ± 39	1355 ± 22	1365 ± 30
Ct.Po% [%]	9 ± 0	8 ± 0	8 ± 0	8 ± 0	12 ± 4	13 ± 3	13 ± 4	11 ± 4
Po.V [mm^3]	0.04 ± 0.01	0.03 ± 0.01	0.04 ± 0.00	0.04 ± 0.00	0.06 ± 0.02	0.06 ± 0.02	0.06 ± 0.02	0.06 ± 0.02
Day 23								
I_{min} [mm^4]	0.04 ± 0.00	0.04 ± 0.00	0.05 ± 0.00	0.05 ± 0.00	0.05 ± 0.01	0.05 ± 0.01	0.04 ± 0.00	0.05 ± 0.01
I_{max} [mm^4]	0.06 ± 0.00	0.06 ± 0.01	0.07 ± 0.01	0.08 ± 0.01	0.08 ± 0.02	0.07 ± 0.02	0.06 ± 0.01	0.08 ± 0.01
Ct.Ar [mm^2]	0.57 ± 0.02	0.57 ± 0.03	0.60 ± 0.03	0.69 ± 0.02	0.61 ± 0.04	0.61 ± 0.03	0.57 ± 0.02	0.69 ± 0.04
Tt.Ar [mm^2]	0.62 ± 0.02	0.61 ± 0.03	0.65 ± 0.03	0.75 ± 0.02	0.69 ± 0.06	0.69 ± 0.06	0.64 ± 0.04	0.76 ± 0.04
Ct.Ar/Tt.Ar [mm^2/mm^2]	0.92 ± 0.00	0.93 ± 0.01	0.92 ± 0.00	0.93 ± 0.01	0.90 ± 0.03	0.89 ± 0.03	0.89 ± 0.03	0.90 ± 0.03
Ct.Th [mm]	0.21 ± 0.01	0.21 ± 0.01	0.22 ± 0.00	0.25 ± 0.01	0.21 ± 0.01	0.21 ± 0.01	0.21 ± 0.01	0.25 ± 0.01
Ct.vTMD [mgHA/cm^3]	1318 ± 11	1334 ± 12	1327 ± 4	1325 ± 19	1363 ± 16	1347 ± 34	1362 ± 20	1360 ± 29
Ct.Po% [%]	8 ± 1	8 ± 1	8 ± 0	8 ± 1	12 ± 4	12 ± 4	12 ± 4	11 ± 4
Po.V [mm^3]	0.04 ± 0.00	0.04 ± 0.00	0.04 ± 0.00	0.04 ± 0.01	0.06 ± 0.02	0.06 ± 0.02	0.06 ± 0.02	0.06 ± 0.02
Day 28								
I_{min} [mm^4]	0.04 ± 0.00	0.04 ± 0.00	0.05 ± 0.00	0.06 ± 0.00	0.05 ± 0.00	0.05 ± 0.00	0.04 ± 0.00	0.05 ± 0.00
I_{max} [mm^4]	0.06 ± 0.00	0.06 ± 0.01	0.07 ± 0.01	0.10 ± 0.01	0.07 ± 0.00	0.07 ± 0.01	0.06 ± 0.00	0.09 ± 0.01
Ct.Ar [mm^2]	0.58 ± 0.02	0.58 ± 0.03	0.61 ± 0.03	0.77 ± 0.03	0.60 ± 0.02	0.60 ± 0.02	0.58 ± 0.02	0.75 ± 0.05
Tt.Ar [mm^2]	0.63 ± 0.02	0.62 ± 0.03	0.66 ± 0.03	0.82 ± 0.03	0.68 ± 0.03	0.67 ± 0.03	0.65 ± 0.03	0.82 ± 0.04
Ct.Ar/Tt.Ar [mm^2/mm^2]	0.92 ± 0.00	0.93 ± 0.00	0.93 ± 0.00	0.94 ± 0.00	0.89 ± 0.03	0.89 ± 0.03	0.89 ± 0.03	0.91 ± 0.03
Ct.Th [mm]	0.22 ± 0.01	0.22 ± 0.01	0.22 ± 0.00	0.28 ± 0.01	0.22 ± 0.01	0.22 ± 0.01	0.21 ± 0.01	0.27 ± 0.02
Ct.vTMD [mgHA/cm^3]	1325 ± 12	1347 ± 10	1339 ± 2	1339 ± 10	1374 ± 17	1378 ± 21	1371 ± 18	1371 ± 17
Ct.Po% [%]	8 ± 1	7 ± 0	8 ± 0	6 ± 0	12 ± 3	12 ± 3	12 ± 4	10 ± 4
Po.V [mm^3]	0.04 ± 0.00	0.04 ± 0.00	0.04 ± 0.00	0.04 ± 0.00	0.06 ± 0.02	0.06 ± 0.02	0.06 ± 0.02	0.06 ± 0.02
Day 33								
I_{min}[a,b,c] [mm^4]	0.04 ± 0.00	0.04 ± 0.00	0.05 ± 0.01	0.06 ± 0.00	0.04 ± 0.00	0.04 ± 0.00	0.04 ± 0.00	0.06 ± 0.01
I_{max}[b,c] [mm^4]	0.06 ± 0.00	0.06 ± 0.01	0.07 ± 0.01	0.10 ± 0.01	0.06 ± 0.00	0.07 ± 0.01	0.06 ± 0.00	0.10 ± 0.01
Ct.Ar[b,c] [mm^2]	0.60 ± 0.02	0.59 ± 0.02	0.63 ± 0.04	0.82 ± 0.03	0.61 ± 0.01	0.60 ± 0.03	0.58 ± 0.02	0.78 ± 0.06
Tt.Ar[b,c] [mm^2]	0.65 ± 0.02	0.63 ± 0.02	0.68 ± 0.04	0.86 ± 0.03	0.68 ± 0.03	0.67 ± 0.04	0.65 ± 0.03	0.85 ± 0.06
Ct.Ar/Tt.Ar[a,c] [mm^2/mm^2]	0.93 ± 0.00	0.93 ± 0.01	0.93 ± 0.01	0.95 ± 0.00	0.90 ± 0.03	0.90 ± 0.02	0.89 ± 0.03	0.92 ± 0.03
Ct.Th[b,c] [mm]	0.23 ± 0.01	0.22 ± 0.01	0.23 ± 0.01	0.29 ± 0.01	0.23 ± 0.02	0.23 ± 0.02	0.22 ± 0.01	0.29 ± 0.02
Ct.vTMD[a] [mgHA/mm^3]	1336 ± 9	1347 ± 11	1348 ± 4	1353 ± 7	1372 ± 26	1385 ± 40	1382 ± 18	1376 ± 15
Ct.Po%[a,c] [%]	8 ± 1	7 ± 1	8 ± 1	5 ± 0	11 ± 4	12 ± 3	12 ± 4	9 ± 4
Po.V[a] [mm^3]	0.04 ± 0.00	0.03 ± 0.00	0.04 ± 0.00	0.04 ± 0.00	0.06 ± 0.02	0.06 ± 0.02	0.06 ± 0.02	0.06 ± 0.02

Table S7: **MicroCT parameters in diaphyseal cortical bone,** the table shows cortical bone parameters in the diaphysis of both limbs on five different experimental days in all experimental groups. Parameters are shown as mean ± standard deviation. *ANOVA main effects:* (a) treatment, (b) loading, (c) limb, post-hoc tests not shown.

	vehicle treated				CC-292 treated			
	nonloaded		loaded		nonloaded		loaded	
	n = 9		n = 8		n = 6		n = 8	
Day 33-13	right limb	left limb	right limb	left limb	right limb	left limb	right limb	left limb
**$\Delta I_{min}^{a,b,c}$ [mm^4]**	0.00 ± 0.00	0.00 ± 0.00	0.00 ± 0.00	0.02 ± 0.00	0.00 ± 0.00	0.00 ± 0.01	0.00 ± 0.01	0.01 ± 0.00
**$\Delta I_{max}^{b,c}$ [mm^4]**	0.00 ± 0.00	0.00 ± 0.00	0.01 ± 0.01	0.04 ± 0.00	0.00 ± 0.01	0.00 ± 0.01	0.00 ± 0.01	0.03 ± 0.01
**ΔCt.Ar^{a,b,c} [mm^2]**	0.04 ± 0.02	0.04 ± 0.02	0.05 ± 0.02	0.24 ± 0.04	0.03 ± 0.02	0.03 ± 0.02	0.03 ± 0.04	0.19 ± 0.05
**ΔTt.Ar^{a,b,c} [mm^2]**	0.04 ± 0.02	0.04 ± 0.02	0.05 ± 0.02	0.23 ± 0.04	0.04 ± 0.03	0.04 ± 0.03	0.03 ± 0.04	0.19 ± 0.05
**ΔCt.Ar/Tt.Ar^{b,c} [mm^2/mm^2]**	0.01 ± 0.01	0.01 ± 0.01	0.01 ± 0.01	0.03 ± 0.01	0.00 ± 0.03	-0.01 ± 0.04	0.00 ± 0.00	0.03 ± 0.01
**ΔCt.Th^{b,c} [mm]**	0.02 ± 0.01	0.02 ± 0.01	0.02 ± 0.01	0.08 ± 0.02	0.02 ± 0.01	0.02 ± 0.02	0.01 ± 0.01	0.07 ± 0.02
**ΔCt.vTMD^{a} [$mgHA/cm^3$]**	36 ± 7	33 ± 9	43 ± 8	44 ± 16	19 ± 22	36 ± 28	35 ± 13	31 ± 22
**ΔCt.Po%^{b,c} [%]**	-1 ± 1	-1 ± 1	-1 ± 1	-3 ± 1	0 ± 3	1 ± 4	-1 ± 0	-4 ± 1
**ΔPo.V [mm^3]**	0.00 ± 0.00	0.00 ± 0.00	0.00 ± 0.00	0.00 ± 0.00	0.00 ± 0.02	0.01 ± 0.02	0.00 ± 0.00	0.00 ± 0.01

Table S8: **Changes in microCT parameters in diaphyseal cortical bone,** the table shows the changes in cortical bone parameters in the diaphysis of both limbs between day 13 and day 33 in all experimental groups. Parameters are shown as mean ± standard deviation. *ANOVA main effects:* (a) treatment, (b) loading, (c) limb, post-hoc tests not shown.

		vehicle treated				CC-292 treated			
		nonloaded		loaded		nonloaded		loaded	
		n = 9		n = 8		n = 6		n = 8	
		right limb	left limb	right limb	left limb	right limb	left limb	right limb	left limb
**Ec.sLS/BS^{a}**	%	0.2 ± 0.1	0.5 ± 0.2	0.2 ± 0.1	0.3 ± 0.2	0.4 ± 0.2	0.5 ± 0.2	0.6 ± 0.3	0.4 ± 0.1
**Ec.dLS/BS^{a}**	%	0.8 ± 0.2	0.6 ± 0.3	0.9 ± 0.2	0.9 ± 0.2	0.6 ± 0.2	0.4 ± 0.3	0.4 ± 0.2	0.5 ± 0.1
**Ec.MS/BS^{a}**	%	0.9 ± 0.1	0.7 ± 0.4	1.0 ± 0.2	1.0 ± 0.1	0.8 ± 0.1	0.6 ± 0.3	0.6 ± 0.2	0.7 ± 0.1
**Ec.MAR(9d)^{a,c}**	(µm/day)	1.6 ± 0.4	1.9 ± 0.6	1.7 ± 0.4	2.1 ± 0.7	1.2 ± 0.4	1.5 ± 0.5	1.2 ± 0.3	1.6 ± 0.5
**Ec.BFR^{a}**	(µm/day)	1.5 ± 0.5	1.7 ± 0.9	1.7 ± 0.4	2.2 ± 0.5	1.0 ± 0.5	1.1 ± 0.6	0.8 ± 0.3	1.2 ± 0.5
**Ps.sLS/BS^{a,c}**	%	0.3 ± 0.1	0.1 ± 0.1	0.2 ± 0.2	0.2 ± 0.1	0.4 ± 0.1	0.4 ± 0.2	0.4 ± 0.2	0.2 ± 0.1
**Ps.dLS/BS^{b,c}**	%	0.3 ± 0.1	0.2 ± 0.2	0.4 ± 0.1	0.6 ± 0.2	0.3 ± 0.2	0.4 ± 0.2	0.1 ± 0.0	0.7 ± 0.1
**Ps.MS/BS^{b,c}**	%	0.3 ± 0.2	0.3 ± 0.2	0.4 ± 0.2	0.8 ± 0.2	0.4 ± 0.2	0.5 ± 0.2	0.2 ± 0.1	0.8 ± 0.1
**Ps.MAR(9d)^{b,c}**	(µm/day)	0.7 ± 0.4	1.2 ± 0.2	1.2 ± 0.5	3.1 ± 0.9	0.8 ± 0.4	1.0 ± 0.3	1.0 ± 0.6	2.8 ± 0.4
**Ps.BFR^{b,c}**	(µm/day)	0.3 ± 0.2	0.4 ± 0.3	0.5 ± 0.2	2.3 ± 1.0	0.3 ± 0.2	0.6 ± 0.2	0.3 ± 0.2	2.3 ± 0.5

Table S9: **Dynamic cortical bone formation indices in the diaphysis** from mice either nonloaded or loaded and treated with vehicle or CC-292. Parameters are shown as mean ± standard deviation. *ANOVA main effects:* (a) treatment, (b) loading, (c) limb, post-hoc tests not shown.
